# Excess mortality of German men and women aged 60 years and older needing long-term care 2020–2024: data from a statutory health insurance comprising more than 3 million people

**DOI:** 10.3389/fpubh.2026.1725110

**Published:** 2026-04-07

**Authors:** Luisa Haß, Danny Wende, Sabrina Voß, Ralph Brinks

**Affiliations:** 1 Faculty of Health/School of Medicine, Herdecke University, Witten, Germany; 2 Department of Health System Research, BARMER Institute for Health System Research, Wuppertal, Germany

**Keywords:** aggregated data, aging, chronic condition, epidemiology, long-term care, mortality, mortality rate ratio

## Abstract

**Introduction:**

Demographic aging is increasing the demand for long-term care (LTC) in Germany. However, existing projections often rely on prevalence-based models that fail to account for age- and sex-specific trends in incidence and mortality. While dynamic approaches, such as the illness-death model, have improved projections, reliable empirical estimates of mortality rate ratios (*MRR*) for LTC recipients are scarce. This study addresses this gap by providing *MRR* estimates based on claims data.

**Methods:**

This study analyzed anonymized, aggregated claims data from BARMER, a German statutory health insurance. The data covered individuals aged 60 years and older from 2020 to 2024. Mortality rates and *MRR* were calculated for individuals with and without LTC needs, stratified by age and sex. Person-time at risk was adjusted assuming mid-year deaths and *MRR* were derived with 95% confidence intervals.

**Results:**

From 2020 to 2024, individuals with LTC need demonstrated persistently higher mortality rates compared to those without LTC need among all age groups and both sexes. The most pronounced increase in mortality disparities was observed in the 90+ age group, where the *MRR* rose from 6.6 in 2020 to 60.7 in 2024 among women and from 5.5 (2020) to 35.2 (2024) among men.

**Conclusion:**

Our analysis reveals a persistent and expanding disparity in mortality among individuals with LTC need across all age groups. The findings indicate that the *MRR*s have remained consistently elevated, suggesting that improvements in survival among individuals with LTC needs have been limited, thereby highlighting their underlying vulnerability.

## 1 Introduction

Health systems worldwide are facing increasing challenges due to demographic aging (1, 2). In Germany, the proportion of older individuals has increased steadily over recent decades, with more than one third of the population aged 65 years or older as of 2022 (3). Concurrent with this development, the number of individuals requiring long-term care (LTC) has also grown continuously. In 2011, approximately 3.4% of those insured under the statutory health insurance system were classified as requiring LTC, compared to 5.9% in 2021 (4). It is expected that these trends will intensify further as the baby boomer generation reaches older age in the coming decades (2).

To estimate future LTC needs, many studies have relied on prevalence-based projections [e.g., (5)]. However, these approaches fail to account for age- and sex-specific trends in incidence and mortality, particularly the differential mortality between individuals with and without LTC needs (6–10). A more recent study has applied the illness-death model (IDM), which provides a more dynamic representation of population transitions between the states “not in need of care,” “in need of care,” and “dead” (7). This projection suggests that the number of people requiring LTC in Germany could more than double by 2050 under various incidence and mortality scenarios (7).

Despite these methodological advances, empirical estimates of mortality rate ratios (*MRR*) for LTC recipients do not exist (7, 11). This gap is concerning, as *MRR* provide a key measure of disease impact in epidemiology (12), enabling quantification of excess mortality among subpopulations as well as estimation of LTC incidence and prevalence.

Previous IDM-based analyses have relied on hypothetical or indirect assumptions about mortality differences between individuals with and without LTC needs. Therefore, this study aims to provide *MRR* estimates for people with and without LTC needs based on claims data from BARMER statutory health insurance.

## 2 Methods

### 2.1 Database

In this analysis, claims data from BARMER, a German statutory health insurance, covering approximately 8.7 million insured individuals is used. However, the data set was restricted to selected age groups (60–90+ years), excluding younger populations for which LTC incidence and mortality rates are either negligible or not the focus of this study. The data set thus comprises anonymized, aggregated information for the years 2020–2024 and includes age- and sex-stratified (male and female) counts of individuals with and without LTC needs, as well as all-cause mortality events.

Throughout the observation period, the total number of individuals included in the analysis ranged from 3.33 million in 2020, 3.49 million in 2021, 3.64 million in 2022, 3.8 million in 2023, to 3.96 million in 2024. Table 1 presents the annual total numbers and proportions of individuals included in the analysis by year, sex and age group.

**Table 1:** Proportion of individuals in need of long-term care (LTC) by year, sex and age group.

Age group	Female			Male		
	LTC	*N*	%	LTC	*N*	%
2020						
60–69 years	34,967	812,505	4.3	27,105	531,289	5.1
70–79 years	85,285	673,731	12.7	48,342	392,251	12.3
80–89 years	198,257	510,813	38.8	80,565	261,967	30.8
90+ years	91,512	112,277	81.5	26,465	38,525	68.7
2021						
60–69 years	38,535	827,927	4.7	29,205	546,391	5.3
70–79 years	88,415	669,140	13.2	49,891	396,082	12.6
80–89 years	222,636	567,176	39.3	88,919	294,624	30.2
90+ years	93,416	136,382	68.5	28,016	49,337	56.8
2022						
60–69 years	41,973	842,742	5.0	31,367	561,719	5.6
70–79 years	94,149	679,604	13.9	52,860	408,046	13.0
80–89 years	243,830	607,268	40.1	96,660	318,436	30.4
90+ years	94,708	162,327	58.3	29,188	61,686	47.3
2023						
60–69 years	46,742	860,548	5.4	34,140	578,898	5.9
70–79 years	100,485	686,108	14.6	56,116	417,710	13.4
80–89 years	267,124	648,947	41.2	105,116	343,364	30.6
90+ years	94,301	190,969	49.4	29,789	75,719	39.3
2024						
60–69 years	51,608	877,596	5.9	36,814	594,423	6.2
70–79 years	105,641	694,599	15.2	59,662	429,761	13.9
80–89 years	283,061	677,496	41.8	110,455	361,307	30.6
90+ years	100,246	231,776	43.3	32,452	96,359	33.7

The classification of LTC need in the underlying data set is based on assessments conducted by the Medical Service (Medizinischer Dienst), whose determinations form the legal and authoritative basis for assigning care grades and, thus, for inclusion in the present analysis.

According to § 14 (1) of Book XI of the German Social Code (SGB XI), individuals are classified as care-dependent if they experience health-related impairments of independence or abilities that necessitate assistance from others. These impairments, which may be physical, cognitive, or psychological, must notably affect the capacity to manage routine activities of daily life. A care-dependent status is recognized only if the condition is expected to persist for a minimum duration of 6 months and meets the severity threshold as defined in § 15 SGB XI.

### 2.2 Statistical analysis

To assess age- and sex-specific mortality rates and mortality rate ratios (*MRR*), the data set for each of the 5 years (2020–2024) was divided into age-groups and stratified by male and female. The analysis was conducted for ages ranging from 60 to 90+ years with 10-year steps. Furthermore, the mortality rates and mortality rate ratios were calculated separately for individuals with and without LTC needs.

To account for differential exposure time due to deaths occurring during the year, it was assumed that individuals, on average, die mid-year (13). Consequently, person-time at risk was adjusted by subtracting half of the number of deaths (*D*) from the population (*N*) at risk. Thus, the mortality rates in person-years (py) for each stratum and year were calculated as follows (13):


$$\frac{D}{(N-0.5\times D)\times 1\;\mathrm{py}}$$

The *MRR* was defined as the ratio of mortality among individuals with LTC need to those without:


$$\mathrm{MRR}=\frac{\text{Mortalit}y_{\mathrm{LTC}}}{\text{Mortalit}y_{\text{NoLTC}}}$$

With a 95% confidence interval (CI) as $\log(\mathrm{MRR})\pm 1.96\cdot SE_{\log(\mathrm{MRR})}$ , where


$$SE_{\log(\mathrm{MRR})}=\sqrt{\frac{1}{D_{\mathrm{LTC}}}+\frac{1}{D_{\text{NoLTC}}}}$$

In addition, a multivariable Poisson regression model with a quasi-Poisson approach was applied to account for potential overdispersion in the data. The dependent variable *D* was modeled as a function of year, sex, as well as the interaction between age group and LTC. Since the research question focused exclusively on estimating age-specific *MRR*s associated with the need for LTC, a model including only the interaction of the age group and LTC was estimated. The main effects of these factors were not added to the model as they are not required for estimating these specific effects and would not meaningfully change the interpretation of the interaction terms. Inclusion of the main effects would make the model more complex without providing additional insight. To account for the time at risk, a log-transformed offset, assuming that deaths occurred on average mid-year, was included. The reference categories in the model were defined as follows: Age group: 60–69 years; LTC: no LTC; Sex: female; Year: 2020. As a result, all reported coefficients represent *MRR* relative to this reference. 95%-CIs were derived using model-based standard errors and exponentiated to facilitate interpretation.

All calculations were performed with the freely accessible, open-source statistical software R Version 4.4.2 (The R Foundation for Statistical Computing).

## 3 Results

Using sex-, age-, and year-specific mortality rates, *MRR* were calculated to compare individuals with LTC need to those without the need for LTC between 2020 and 2024. Overall, the findings indicate a consistently and substantially elevated mortality rate among LTC-dependent individuals compared to those without, across all years and age groups, irrespective of the individual’s sex.

**Figure 1:**
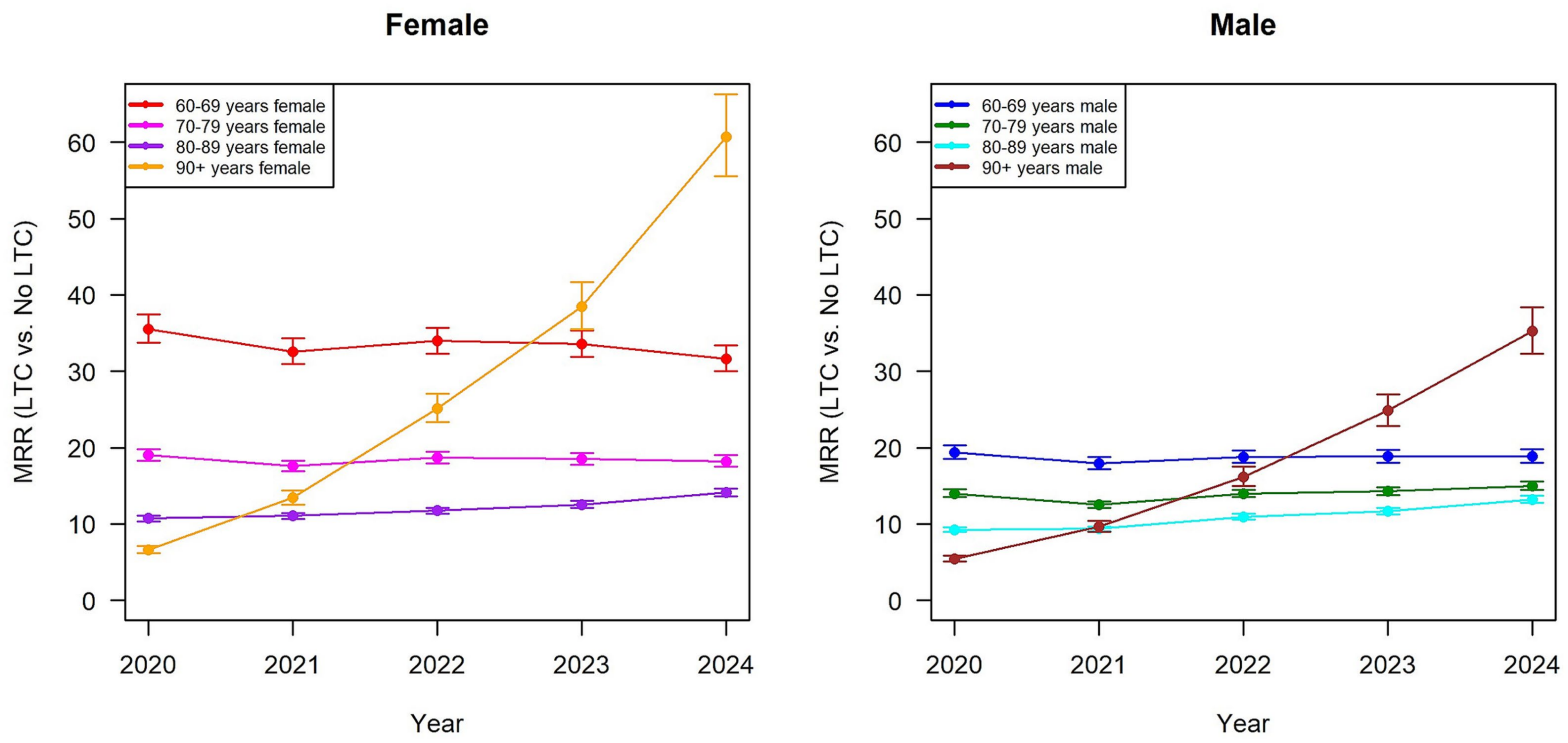
Mortality rate ratio (MRR) of people with and without long-term care (LTC) needs from 2020 to 2024, stratified by sex (women left panel, men right panel) and four different age groups.

The contrast between the beginning and the end of the observation period is particularly striking, especially with regard to mortality disparities between individuals with and without LTC need (see Figure 1).

For instance, among women aged 90 years and older, the mortality rate without the need for LTC was 0.039, compared to 0.256 with the need for LTC (see Table 2), resulting in a *MRR* of 6.6 (95%-CI: 6.15–7.08). Similarly, for men in the same age group, mortality was 0.061 without LTC and 0.334 with LTC (*MRR* = 5.45; 95%-CI: 5.05–0.5.88) (see Table 3). In both cases, the CIs were relatively narrow, indicating limited statistical uncertainty in the estimates. By 2024, the mortality rate for individuals without LTC in this age group has decreased, while mortality among those with LTC need remained relatively stable. This resulted in an increase in the *MRR* to 35.2 (95%-CI: 32.3–38.4) and 60.7 (95%-CI: 55.6–66.3) for men and women, respectively (see Figure 1; Tables 2, 3), with both CIs suggesting more uncertainty around the estimate. A similar, albeit less dramatic, pattern was observed in the 80–89 years age group for both sexes (see Figure 1; Tables 2, 3). The *MRR* of the age group of people aged 70–79 years, however, remained relatively stable over time (see Figure 1; Tables 2, 3). This pattern of stability was also evident among males aged 60–69, with the *MRR* remaining nearly unchanged from 19.4 in 2020 to 18.9 in 2024 (see Figure 1; Table 3). Among females in the same age group, the *MRR* decreased slightly from 35.5 to 31.7, though remaining high, for 2020 and 2024, respectively (see Figure 1; Table 2).

**Table 2:** Mortality rates and mortality rate ratios (MRR) among female individuals with and without long-term care (LTC) needs from 2020 to 2024 for the age groups 60–69, 70–79, 80–89, 90+ years.

Age group	Mortality rate		MRR	95%-CI
	LTC	No LTC		
2020				
60–69 years	0.104	0.003	35.535	33.71–37.46
70–79 years	0.112	0.006	19.018	18.29–19.78
80–89 years	0.128	0.012	10.729	10.36–11.11
90+ years	0.256	0.039	6.599	6.15–7.08
2021				
60–69 years	0.101	0.003	32.594	30.97–34.3
70–79 years	0.107	0.006	17.565	16.9–18.26
80–89 years	0.122	0.011	11.052	10.68–11.44
90+ years	0.249	0.019	13.421	12.5–14.41
2022				
60–69 years	0.100	0.003	33.954	32.27–35.73
70–79 years	0.106	0.006	18.693	17.97–19.45
80–89 years	0.125	0.011	11.729	11.34–12.13
90+ years	0.267	0.011	25.125	23.32–27.07
2023				
60–69 years	0.091	0.003	33.546	31.85–35.33
70–79 years	0.099	0.005	18.532	17.8–19.3
80–89 years	0.114	0.009	12.551	12.12–13.0
90+ years	0.252	0.007	38.484	35.55–41.66
2024				
60–69 years	0.081	0.003	31.666	30.04–33.38
70–79 years	0.090	0.005	18.218	17.47–18.99
80–89 years	0.110	0.008	14.143	13.63–14.68
90+ years	0.234	0.004	60.721	55.59–66.32

**Table 3:** Mortality rates and mortality rate ratios (MRR) among male individuals with and without long-term care (LTC) needs from 2020 to 2024 for the age groups 60–69, 70–79, 80–89, 90+ years.

Age group	Mortality rate		MRR	95%-CI
	LTC	No LTC		
2020				
60–69 years	0.147	0.008	19.392	18.54–20.29
70–79 years	0.185	0.013	14.001	13.5–14.52
80–89 years	0.221	0.024	9.248	8.94–9.56
90+ years	0.334	0.061	5.451	5.05–5.88
2021				
60–69 years	0.140	0.007	17.969	17.19–18.78
70–79 years	0.173	0.014	12.522	12.08–12.98
80–89 years	0.211	0.023	9.365	9.06–9.68
90+ years	0.331	0.034	9.678	8.97–10.45
2022				
60–69 years	0.137	0.007	18.814	18.0–19.66
70–79 years	0.178	0.013	13.996	13.5–14.51
80–89 years	0.216	0.020	10.955	10.6–11.32
90+ years	0.345	0.021	16.188	14.98–17.5
2023				
60–69 years	0.127	0.007	18.824	18.0–19.68
70–79 years	0.163	0.011	14.305	13.78–14.85
80–89 years	0.198	0.017	11.688	11.3–12-09
90+ years	0.325	0.013	24.834	22.86–26.98
2024				
60–69 years	0.115	0.006	18.874	18.03–19.76
70–79 years	0.153	0.010	15.010	14.44–15.6
80–89 years	0.186	0.014	13.224	12.76–13.71
90+ years	0.303	0.009	35.233	32.31–38.41

The regression results were consistent with the descriptive findings. Across all age groups, individuals with LTC need showed markedly higher mortality compared to those without LTC need (see Table 4). The *MRRs* increased with age, ranging from 24.2 (95% CI: 20.4–28.8) among individuals aged 60–69 to 65 (95% CI: 56.5–75.2) among those aged 90 years and older both compared to the reference category (age 60–69 years, without LTC need) (see Table 4). This shows that older people with LTC had a substantially higher mortality. If considering only the effect of sex, mortality was 66% higher among men (*MRR* = 1.66; 95% CI: 1.57–1.76) compared to women in the same year and with the same interaction of age and LTC (see Table 4).

**Table 4:** Exponentiated coefficients from the quasi-Poisson regression model estimating mortality rate ratios (MRR) by year, sex, age group, and long-term care (LTC) need compared to the reference category (60–69 years, no LTC, female, 2020).

Variable	MRR	95%-CI	SE	*p* < 0.05
2021	0.965	0.88–1.06	0.047	0.455
2022	0.97	0.88–1.06	0.046	0.507
2023	0.886	0.81–0.97	0.047	0.012*
2024	0.82	0.75–0.90	0.047	0.000*
Sex	1.662	1.57–1.76	0.030	0.000*
90+ years with LTC	65.028	56.51–75.16	0.073	0.000*
80–89 years with LTC	33.84	29.55–38.94	0.070	0.000*
70–79 years with LTC	28.57	24.63–33.26	0.077	0.000*
60–69 years with LTC	24.185	20.37–28.76	0.088	0.000*
90 + years without LTC	2.986	2.18–4.01	0.155	0.000*
80–89 years without LTC	3.001	2.53–3.57	0.088	0.000*
70–79 years without LTC	1.803	1.52–2.15	0.088	0.000*

*Significant.

With respect to temporal patterns, the model showed no statistically significant decreases in the mortality in 2021 and 2022 compared to 2020, while mortality decreased significantly in 2023 with a 11% reduction (*MRR* = 0.89; 95% CI: 0.81–0.97) and in 2024 with a 18% reduction (*MRR* = 0.82; 95% CI: 0.75–0.90) (see Table 4) for people with the same LTC status, sex and at the same age. These findings align with the descriptive trends presented above (see Figure 1; Tables 2, 3).

## 4 Discussion

### 4.1 Summary

To our knowledge, this analysis was the first to provide a detailed, stratified calculation of mortality trends of LTC in Germany using aggregated claims data. This analysis investigated mortality trends from 2020 to 2024 among individuals aged 60 years and older, stratified by age, sex, and LTC need. Mortality rates were calculated separately for people with and without LTC and *MRR* were used to assess the relative mortality risk associated with LTC need.

Our findings highlight pronounced and persistent mortality differences between individuals with and without LTC need. However, they also reveal heterogeneities by sex and age-group. Throughout all years and subgroups, individuals with LTC need consistently experienced significantly higher mortality rates than those without. While mortality rates among people without LTC need steadily decreased during the five-year period, mortality rates among people with LTC need showed less consistent changes—either increasing dramatically, remaining stable or fluctuating slightly depending on age and sex (see Tables 2, 3).

The regression model showed that men had a 66% higher overall mortality risk than women, when adjusting for all covariates but sex (see Table 4). However, the descriptive LTC-specific *MRR*s were higher among women (see Table 2), which is reflected by the lower mortality among women without LTC need; consequently, increases in mortality rate among women with LTC need translate into higher *MRR*s.

At the descriptive level, the *MRR* in the oldest age group (90 + years) increased substantially over the study period—from 6.6 to 60.7 in women, and from 5.5 to 35.2 in men (see Tables 2, 3)—representing an approximate nine-fold and six-fold increase, respectively. This is mainly driven by declining mortality among individuals without LTC need rather than improvements among those with LTC need. Notably, individuals aged 60–69 years showed significantly elevated *MRR*s throughout the entire study period (around 35 for women and 19 for men [see Tables 2, 3)]. Conversely, *MRR*s in the 70–79 and 80–89 year groups demonstrated an increase, albeit to a lesser extent than the youngest cohort.

### 4.2 Limitations

Certain limitations should be considered when interpreting the results of this analysis.

The analysis is based on claims data from a single statutory health insurance (BARMER). While this data set offers rich and detailed information on LTC status and mortality, it may not fully capture individuals insured by other statutory or private health insurances in Germany. The findings may thus not be fully generalizable to the whole German population. Confounding factors such as comorbidities, socioeconomic status, or access to healthcare could influence the observed trends and were not explicitly adjusted for in the present analysis.

While this data set offers valuable insights into LTC need and mortality, it is important to note that insured populations may vary systematically across different statutory health insurances and also compared to privately insured individuals in Germany. Prior studies have demonstrated that the profiles of insured individuals differ (14), however, as Breslow and Day (15), p. 59 noted, absolute risks may vary across different insurances, yet relative risks are expected to remain stable across a broad range of populations.

A further limitation could be the completeness of the available data. In Germany, mortality events in statutory health insurance data are typically very complete. This is due to the fact that insurance membership formally ends at the time of death, and insurers routinely receive official notifications for administrative updates. Individuals with incomplete insurance periods were excluded from the analyses to mitigate potential distortions. In this particular instance, it was hypothesized that any individual who transitioned between health insurance providers was still alive and no longer subject to observation. Therefore, even in the event of minor discrepancies in mortality documentation, the resulting bias is likely to reduce the *MRR* toward the null effect (*MRR* = 1) rather than inflate it.

Another potential constraint of the data set is that the annual counts of individuals might not accurately reflect unique persons across the study period, as individuals could be included in multiple years. Consequently, the reported totals (see Table 1) should be interpreted as year-specific rather than cumulative population figures.

Additionally, the data used in this study covers the period of the COVID-19 pandemic, during which there may have been temporary shifts in mortality patterns. Accordingly, all identified trends should be regarded as potential tendencies.

Moreover, LTC need was defined based on § 14 (1) of Book XI of the German Social Code (SGB XI) and determination of LTC need in the underlying data set is based on assessments conducted by the Medical Service. However, this measure depends on the application and assessment process, which may vary over time and between individuals. People with LTC needs might remain unclassified if they do not apply or face barriers to access. Additionally, alterations in legislation and administrative practices have the potential to impact the consistency of LTC status over time. For instance, the 2017 reform introduced a novel, more inclusive definition of LTC need, whereby earlier care levels were incorporated into care grades (16). Consequently, the continued modifications may result in system-related increases of the LTC prevalence.

Moreover, LTC need inherently captures advanced morbidity, functional decline, and frailty. The estimated *MRRs* therefore do not represent causal effects of LTC certification itself but rather reflect the mortality associated with this legally defined state of severe and long-lasting health impairment. LTC need should therefore be interpreted as a summary marker of accumulated vulnerability rather than an independent risk factor. Apart from the data limitations mentioned, further uncertainties may arise due to estimation of *MRR* with aggregated data. This approach does not control for individual-level factors such as the place of residence (i.e., inpatient, outpatient, formal or informal care), disease burden, frailty, or socioeconomic characteristics which may lead to unmeasured heterogeneity in mortality rates.

In order to adjust for differing exposure times resulting from within-year deaths, it was assumed that individuals, on average, died halfway through the year (sensitivity analyses see Supplementary Tables S1, S2). While this approach is common, it relies on the assumption of deaths being evenly distributed over the year. However, this may not always be the case, e.g., during times of extraordinary events.

In addition, as previously indicated by Haß et al. (11), the remission rate was assumed to be zero. Theoretically, individuals with LTC need may regain their autonomy and independence, depending on the presence and severity of comorbidities. However, such cases are comparatively uncommon in contrast to care termination due to death (17).

Despite these limitations, this analysis represents the first population-based estimation of mortality differences in Germany between people in need for LTC and those without using data from a large statutory health insurance.

### 4.3 Literature comparison

As far as the authors are aware, empirical estimates of *MRR* comparing individuals with and without LTC needs in Germany do not yet exist. While prior studies have reported excess mortality among older adults during the COVID-19 pandemic (18, 19) and elevated mortality in LTC populations both before and during the pandemic (20, 21), they did not provide quantification of *MRR*. Our analysis addresses this gap by presenting the first comprehensive *MRR* estimates for older adults with LTC needs over the years 2020–2024.

### 4.4 Conclusion/outlook

The findings of this analysis highlight a persistent and growing disparity in mortality between individuals with and without LTC need. This considerable disparity may, at least in part, reflect the exceptional vulnerability of the individuals during the COVID-19 pandemic, particularly among the oldest individuals with LTC need. Among individuals aged 60–69 years, the *MRR*s were also remarkably elevated, suggesting that the requirement of LTC at comparatively younger ages might be associated with a particularly severe underlying health burden, resulting in a significantly stronger relative mortality disadvantage. Overall, the high *MRR*s suggest that people with LTC need remain a highly vulnerable population with limited improvements in survival over time.

This study provides an important initial foundation for further research. Future studies should build on these findings to refine *MRR* over time and apply them to calculate more precise measures of annual percent changes and incidence of LTC. Such improvements will ultimately allow for more accurate LTC projections of the number of care-dependent individuals and inform evidence-based health and social policy planning.

## Data Availability

The datasets presented in this study can be found in online repositories. The names of the repository/repositories and accession number(s) can be found at: https://zenodo.org/records/17877557.
