# Supplementary material for: Excess mortality of German men and women aged 60 years and older needing long-term care 2020–2024: data from a statutory health insurance comprising more than 3 million people
Source: Front Public Health. 2026 Apr 7;14:1725110. doi: 10.3389/fpubh.2026.1725110 (PMC13095577; doi:10.3389/fpubh.2026.1725110)
Supplement: Supplementary file 1 [file Table_1.docx]

**Supplementary Files**

***Table S1:*** *Exponentiated coefficients (mortality rate ratios) from a quasi-Poisson regression model estimating age-, sex-, year-, and LTC-specific mortality under alternative assumptions regarding the timing of death within the calendar year (0.45), shown relative to the reference category (age 60–69 years, no LTC, female, 2020).*

| **Variable** | **MRR** | **95%-CI** | **SE** | **p < 0.05** |
| --- | --- | --- | --- | --- |
| 2021 | 0.965 | 0.88-1.06 | 0.047 | 0.459 |
| 2022 | 0.97 | 0.89-1.06 | 0.047 | 0.508 |
| 2023 | 0.887 | 0.81-0.97 | 0.047 | 0.013* |
| 2024 | 0.82 | 0.75-0.90 | 0.047 | 0.000* |
| Sex | 1.66 | 1.56-1.76 | 0.030 | 0.000* |
| 90+ years with LTC | 64.1 | 55.7-74.1 | 0.073 | 0.000* |
| 80-89 years with LTC | 33.6 | 29.3-38.7 | 0.071 | 0.000* |
| 70-79 years with LTC | 28.4 | 24.5-33.0 | 0.077 | 0.000* |
| 60-69 years with LTC | 24.1 | 20.2-28.6 | 0.088 | 0.000* |
| 90+ years without LTC | 2.98 | 2.18-4.01 | 0.155 | 0.000* |
| 80-89 years without LTC | 3.00 | 2.52-3.57 | 0.088 | 0.000* |
| 70-79 years without LTC | 1.80 | 1.52-2.15 | 0.089 | 0.000* |

** = significant*

***Table S2****: Exponentiated coefficients (mortality rate ratios) from a quasi-Poisson regression model estimating age-, sex-, year-, and LTC-specific mortality under alternative assumptions regarding the timing of death within the calendar year (0.55), shown relative to the reference category (age 60–69 years, no LTC, female, 2020).*

| **Variable** | **MRR** | **95%-CI** | **SE** | **p < 0.05** |
| --- | --- | --- | --- | --- |
| 2021 | 0.965 | 0.88-1.06 | 0.047 | 0.45 |
| 2022 | 0.97 | 0.89-1.06 | 0.046 | 0.508 |
| 2023 | 0.886 | 0.81-0.97 | 0.047 | 0.013* |
| 2024 | 0.82 | 0.75-0.90 | 0.047 | 0.000* |
| Sex | 1.67 | 1.57-1.77 | 0.030 | 0.000* |
| 90+ years with LTC | 66.0 | 57.3-76.2 | 0.073 | 0.000* |
| 80-89 years with LTC | 34.1 | 29.8-39.2 | 0.070 | 0.000* |
| 70-79 years with LTC | 28.8 | 24.8-33.5 | 0.076 | 0.000* |
| 60-69 years with LTC | 24.3 | 20.5-28.9 | 0.088 | 0.000* |
| 90+ years without LTC | 2.99 | 2.18-4.01 | 0.155 | 0.000* |
| 80-89 years without LTC | 3.00 | 2.53-3.57 | 0.088 | 0.000* |
| 70-79 years without LTC | 1.80 | 1.52-2.15 | 0.088 | 0.000* |

** = significant*
